# Investigation of Structural, Elastic and Magnetic Properties of CoCr_2−x_Zr_x_O_4_ Nanoparticles

**DOI:** 10.3390/ma17215149

**Published:** 2024-10-22

**Authors:** Mai M. E. Barakat, Doaa El-Said Bakeer

**Affiliations:** 1 Department of Physics, Faculty of Science, Taibah University, Yanbu 46423, Saudi Arabia; 2 Department of Physics, Faculty of Science, Alexandria University, Alexandria 21568, Egypt; 3 Department of Physics, Faculty of Science, Damanhour University, Damanhour 22511, Egypt

**Keywords:** cobalt chromates, elastic properties, FTIR, VSM, co-precipitation

## Abstract

This study investigates the impact of zirconium substitution on the structural, elastic and magnetic properties of CoCr_2_O_4_ nanoparticles. A series of CoCr_2−x_Zr_x_O_4_ nanoparticles, x = 0.00, 0.05, 0.10, 0.15 and 0.20, are synthesized via the co-precipitation method. X-ray diffraction (XRD) patterns affirm the formation of single-phase cubic structure with the space group Fd3m. Special attention is given to accurately calculating the average crystallite size (*D*) and lattice parameter (*a*) using Williamson–Hall (W–H) analysis and the Nelson–Riley (N–R) extrapolation function, respectively. The increase in Zr^4+^ content leads to a reduction in crystallite size and an increase in the lattice parameter. Elastic properties are estimated from force constants and the lattice constant, determined from FTIR and XRD, respectively. The observed changes in the elastic constants are attributed to the strength of interatomic bonding. The stiffness constants decrease, while Poisson’s ratio increases with increasing Zr^4+^ content, reflecting the increase in the ductility of the prepared samples. As the Zr^4+^ content increases, the stiffness constants decrease, and Poisson’s ratio increases, reflecting enhanced ductility of the samples. Furthermore, as Zr^4+^ content rises, Young’s modulus, the rigidity modulus and Debye temperature decrease. The magnetic hysteresis loop measurements are carried out at room temperature using a vibrating sample magnetometer (VSM) over a field range of 25 kg. Unsubstituted CoCr_2_O_4_ exhibits ferrimagnetic behavior. As Zr^4+^ content increases, saturation magnetization (*M_s_*) and magnetic moment decrease, while remanent magnetization (*M_r_*) and coercivity (*H_c_*) initially decrease up to x = 0.10, then increase with further increases in x. The novel key of this study is how Zr^4+^ substitution in CoCr_2_O_4_ nanoparticles can effectively modify their elastic moduli and magnetic properties, making them suitable for various applications such as flexible electronics, protective coatings, energy storage components and biomedical implants.

## 1. Introduction

Chromates (MCr_2_X_4_; M = Co, Fe, etc., X = O, S ions) are members of the multiferroic materials class, exhibiting uniform magnetization as huge ferrimagnetism, spatially modulated magnetism and magneto resistance [1,2]. The normal cubic spinel structure for cobalt chromites, CoCr_2_O_4_, arises from the occupation of tetrahedral sites by Co^2+^ divalent cations and octahedral sites by Cr^3+ +^ trivalent cations [3,4,5,6]. Three magnetic transitions are sustained by CoCr_2_O_4_ as a result of the exchange interaction competing for Cr–Cr, Co-Cr and Co–Co. (1) The existence of long-range non-collinear spiral and ferroelectric magnetic transition occurs at *T_S_* ≈ 26 K. (2) Above *T_S_*, the material undergoes a transition from paramagnetic to collinear short-range ferrimagnetism, reaching Curie temperature *T_C_* ≈ 95 K. CoCr_2_O_4_ has a multiferroic property, resulting from the onset of non-collinear spiral magnetic transition to the onset of shortrange ferrimagnetic transition [7,8]. (3) Below *T_S_*, a lock-in transition appears at *T*_L_ ≈ 14 K. Both ferromagnetic and ferroelectric orders give cobalt chromates great attention in practical applications such as high temperature ceramics, catalysis, semiconductors, biomedical materials, electrochemical sensors, telecommunication systems, data storage media and various nanotechnology technological applications [9,10,11,12,13,14,15].

The properties of magnetic materials, such as permanent magnetization and magnetic saturation, are strongly influenced by particle size. Therefore, the preparation methods of magnetic materials play an important role in controlling the particle size and shape. Several methods have been used to prepare CoCr_2_O_4_ nanoparticles, such as the hydrothermal method [16], the sonochemical technique [17], thermolysis of the polymer metal complex [18,19], co-precipitation [20], the low-temperature combustion method by citric acid [21,22] and the sole gel route with propylene oxide as a gelation agent [23]. Among all these techniques, the co-precipitation method stands out for nanoparticle synthesis due to its simplicity, cost-effectiveness and scalability. It offers precise control over particle size, shape and composition by adjusting reaction conditions, producing uniform and high-purity nanoparticles. Its versatility allows for the synthesis of a wide range of materials, with the potential for doping and functionalization. Additionally, co-precipitation delivers reproducible results, making it suitable for both industrial and research applications in catalysis, biomedical fields and environmental remediation. The average particle size of CoCr_2_O_4_ ranges from 8 to 40 nm, depending on the preparation method and combustion or annealing temperatures [17,18,19,20,21,22,23]. The hydrothermal preparation for CoCr_2_O_4_ tends to produce particle sizes greater than 90 nm [16].

The effect of the partial ionic substitution for CoCr_2_O_4_ nanoparticles brings great attention to different electrical and magnetic applications [24]. Modifying the structural and magnetic properties of CoCr_2_O_4_ nanoparticles can be achieved through changing the dopant nature, dopant concentration, sintering temperature and dopant particle size. The increase in Mg concentration into Co_1−x_Mg_x_Cr_2_O_4_ nanoparticles suppresses both *T_C_* and *T_S_* transition states [25]. The decrease in *T_C_* value was reported with increasing Zn concentration into Co_1−x_Zn_x_Cr_2_O_4_ nanoparticles [26]. The effects of various dopants on Co_1−x_M_x_Cr_2_O_4_ (M = Zn, Cu, Mg) were studied by Pankaj and Dinesh [27], who found a change in the structure of CoCr_2_O_4_ nanoparticles with different doping. Moreover, the doping with Zn and Mg made CoCr_2_O_4_ nanoparticles more suitable for high frequency microelectronic applications in comparison with those doped by Cu. The increase in Mn doping into Co_1−x_Mn_x_Cr_2_O_4_ nanoparticles resulted in lower *T_C_*, *T_S_* and *T_L_* values but enhanced the saturation magnetization [28]. The temperature-dependent magnetic response of CoCr_2_O_4_ nanoparticles warrants emphasis [29,30,31]. Researchers have demonstrated that the ZFC/FC curves indicate a transition from paramagnetic to ferromagnetic behavior at T_c_ = 100 K. As the temperature decreased, the magnetization ($M_{s}$) reached a maximum at 75 K before gradually declining to 5 K [29]. Additionally, a sharp increase in coercivity (H_C_) at low temperatures was observed, consistent with the modified Kneller’s law [29].

Numerous studies have examined the impact of Zr^4+^ ion substitution on the structural and physical properties of oxide nanomaterials [32,33,34]. Kavitha et al. [32] reported that in Co_1−x_Zr_x_Fe_2_O_4_, well-defined reflections of spinel lattice planes were observed at lower zirconium concentrations. However, at high Zr content for x ≥ 0.75, an impurity peak corresponding to Fe_2_O_3_ appears. Additionally, the (311) peaks shifted slightly to higher angles, indicating strain induced by the larger Zr ions due to ionic size mismatch. Naik et al. [33] showed that Zn_1−x_Zr_x_O (0 ≤ x ≤ 0.05) nanoparticles showed no impurity phase in both undoped and Zr-doped ZnO. Monaji et al. [34] confirmed that for Co_1+x_Zr_x_Co_2−2x_O_4_ (0 ≤ x ≤ 0.4), X-ray spectral analysis revealed a single-phase cubic structure up to x = 0.2 with no impurity phases. In addition, coercivity ($H_{c}$) was found to increase significantly with decreasing temperature for all Zr-substituted samples. Based on the earlier findings, it is expected that substituting with a high concentration of Zr^4+^ ions could destabilize the structure, potentially leading to the formation of secondary phases and introducing strain due to the larger size of the Zr ions. The substitution of Zr in BaZ_x_Ti_1−x_O_3_ enhanced the chemical stability and reduced the dielectric loss [35]. The magnetic properties of Mg_0.5_Cu_0.5_Fe_2−2x_Ni_x_Zr_x_O_4_ were significantly affected by the Zr^4+^ dopant, as it weakened the material’s magnetic properties [36]. Reda et al. [37] reported that doping BaTi_1_₋_x_Zr_x_O_3_ with Zr greatly improved its ferroelectric properties. Furthermore, a decrease in the energy band gap was observed in LaFe_1−x_Zr_x_O_3_ as the Zr content increased [38].

FTIR and the elastic properties of solids are linked through molecular vibrations and structural characteristics. FTIR measures infrared radiation absorption, revealing a material’s molecular composition and bonding. These molecular features significantly influence elastic properties, such as Young’s modulus and shear modulus. By analyzing FTIR spectra, researchers can deduce how molecular interactions affect physical properties, including force constants, elastic moduli, Debye temperature and molar heat capacity [39,40,41,42].

This study mainly focuses on synthesizing CoCr_2−x_Zr_x_O_4_ nanoparticles through the best conditions of the co-precipitation technique and studying the effects of Zr^4+^ substitution on the structural, elastic and magnetic properties of cobalt chromates nanoparticles. It is important to note that a comprehensive study on the structural, elastic and magnetic properties of Zr^4+^ substituted CoCr_2_O_4_ nanoparticles has not yet been reported. The elastic properties of cobalt chromates are a powerful tool for determining its suitability for industrial applications. Furthermore, understanding the elastic behavior is crucial for gaining insights into the thermal properties of chromates and revealing the nature of their interionic bonding. FTIR spectroscopy is often used to estimate the elastic properties of such materials.

## 2. Experimental Techniques

A series of CoCr_2−x_Zr_x_O_4_ nanoparticles with varying zirconium content (x = 0.00, 0.05, 0.10, 0.15 and 0.20) were synthesized using a chemical co-precipitation method. Stoichiometric amounts of analytical-grade chemical reagents, cobalt chloride hexahydrate (CoCl_2_·6H_2_O, Sigma-Aldrich, St. Louis, MO, USA, ≥98%), chromium chloride hexahydrate (CrCl_3_·6H_2_O, Sigma-Aldrich, ≥98%) and zirconium chloride octahydrate (ZrOCl_2_·8H_2_O, Sigma-Aldrich, 98%) were dissolved in distilled water to form homogeneous aqueous solutions. The solutions were thoroughly mixed using a magnetic stirrer and heated to 40 °C (Ceramic Plate LCD Digital Magnetic Hotplate Stirrer, Klang, Selangor, Malaysia). Sodium hydroxide (NaOH) was then added to the salt solution to adjust the pH to 12.0, resulting in the formation of a precipitate. The mixture was continuously stirred and heated to 60 °C, where it was maintained for 2 h. The precipitate was washed with deionized water to remove any residual salts and then dried at 100 °C for 24 h to eliminate any remaining water. Finally, the dried sample was calcined at 600 °C for 2 h to enhance its crystalline properties.

The structural and phase purity of CoCr_2−x_Zr_x_O_4_ nanoparticles was analyzed using X-ray powder diffraction (XRD) with a Bruker D8 Advance powder diffractometer (Billerica, MA, USA), utilizing Cu-Kα radiation (λ = 1.54056 Å) over 2θ ranges from 10° to 80°. The morphology and size of the synthesized nanoparticles were examined using a Jeol JEM-2100 transmission electron microscope (TEM) operating at 200 kV (Tokyo, Japan). Elemental composition of cobalt chromate was determined via scanning electron microscopy with energy-dispersive X-ray spectroscopy (SEM/EDS; JSM-7200F). Fourier transform infrared (FTIR) spectroscopy was conducted on a Shimadzu FTIR 8400S spectrophotometer with KBr pellets (Kyoto, Japan). The magnetic properties of the CoCr_2−x_Zr_x_O_4_ nanoparticles (0.00 ≤ x ≤ 0.20) were evaluated at room temperature using a Lakeshore 7410 vibrating sample magnetometer (VSM) (Westerville, OH, USA).

## 3. Results and Discussion

**Figure 1:**
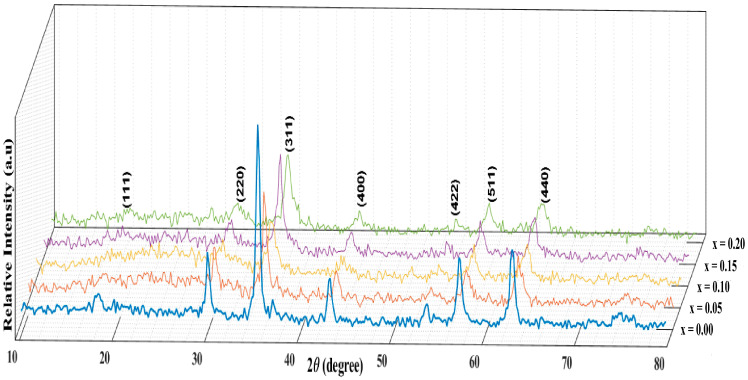
XRD patterns of CoCr_2−x_Zr_x_O_4_ nanoparticles, 0.00 ≤ x ≤ 0.20.

To study the effect of Zirconium substitution on the phase formation and the crystallite size of CoCr_2_O_4_, XRD analysis is performed on the prepared samples. Figure 1 illustrates the XRD patterns for CoCr_2−x_Zr_x_O_4_, where x varies from 0.0 to 0.20. The prepared samples display well-defined Bragg reflection planes, indicating robust crystallization. Structural analysis affirms a single-phase cubic spinel structure (JCPDS card 001-1122) [43] with the space group Fd3m, devoid of additional diffraction lines associated with other crystallographic phases or unreacted components. Previous works reported similar behavior, where no impurity phases appeared with increasing Zr^4+^ content in other oxide nanomaterials [32,33,34]. As observed in Figure 1, the diffraction peaks of CoCr_2_O_4_ spinel phase broaden and lose sharpness with increasing Zr^4+^ content, leading to a decrease in crystallite size.

The experimentally determined lattice parameter, $a_{exp}$, is calculated using the following formula:

(1) $$a_{exp}=d_{hkl}{\left[h^{2}+k^{2}+l^{2}\right]}^{1/2}$$

where *d_hkl_* is plane spacing, and *h*, *k* and *l* are Miller indices.

The theoretical lattice parameter $a_{th}$ of the investigated samples is calculated using the following equation [44]:

(2) $$a_{th}=\frac{8}{3\sqrt{3}}\left[\left(r_{a}+R_{0}\right)+\sqrt{3\;}\;(r_{b}+R_{0})\right]$$

where $R_{0}$ is the radius of the oxygen ion (1.32 Å), and $r_{a}$ and $r_{b}$ are the ionic radii of the tetrahedral and octahedral sites, respectively. Table 1 clearly demonstrates a match between the theoretical and experimental lattice parameters, confirming the expansion of the lattice.

The Nelson–Riley (N–R) method [45] provides a precise determination of lattice parameters through the following function:

(3) $$F\left(\theta \right)=\frac{1}{2}\left[\left(\frac{{Cos}^{2}\theta }{Sin\theta }\right)+\left(\frac{{Cos}^{2}\theta }{\theta }\right)\right]$$

**Figure 2:**
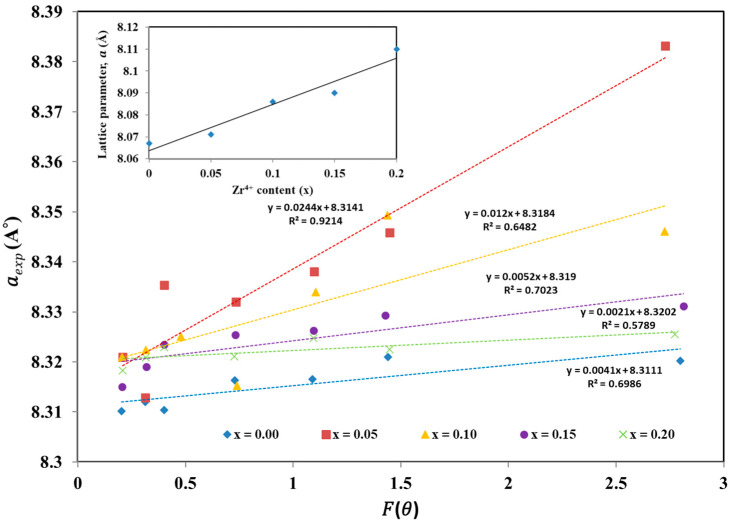
The variation of lattice parameter vs. Nelson–Riley (N–R) function for CoCr_2−x_Zr_x_O_4_ nanoparticles, 0.00 ≤ x ≤ 0.20. The inset shows the variation of lattice parameter with Zr^4+^ content, x.

Figure 2 displays the linear fit of $a_{exp}$ versus $F\left(\theta \right)$ for the prepared samples. A graph is plotted with the calculated values of lattice parameters for each peak of a sample against $F\left(\theta \right)$. By extrapolating these plots at $F\left(\theta \right)\to 0$, accurate values of the lattice constant are obtained, as listed in Table 1. The inset of Figure 2 illustrates that the lattice parameter adheres to Vegard’s law [46], showing a linear increase with the rise in Zr^4+^ content. This increase is attributed to the larger ionic radius of Zr^4+^ (0.86 Å) compared to Cr^3+^ (0.755 Å), with both ions having a coordination number of 6.

The percentage porosity (P%) of the samples is estimated using the following relation [47]:

(4) $$P\%=\left(1-\frac{\rho_{b}}{\rho_{x}}\right)\times 100$$

where $\rho_{b}$ and $\rho_{x}$ are the experimental and theoretical (X-ray) densities, respectively.

(5) $$\rho_{x}=\frac{ZM_{wt}}{N_{A}a^{3}}$$

(6) $$\rho_{b}=\frac{m}{{\pi r}^{2}t}$$

where Z is the number of molecules per primitive unit cell (Z = 8), $M_{wt}$ is the molecular weight of the material, *N_A_* is Avogadro’s number and m, r and t are the mass, radius and thickness of the pellet, respectively.

As observed in Table 1, the value of $\rho_{x}$ for CoCr_2_O_4_ is consistent with the value reported in literature [48]. The X-ray density ($\rho_{x}$) increases with higher Zr^4+^ content. This increase in $\rho_{x}$ is likely due to the rise in molecular weight ($M_{wt}$) associated with increasing Zr^4+^ content, which outweighs the effect of the expanding lattice constant. Additionally, $\rho_{x}$ is higher than the bulk density ($\rho_{b}$) due to the presence of pores, which are influenced by both the sintering temperature and the preparation conditions [49,50]. The inverse behavior between the porosity and $\rho_{x}$ is observed, as it decreases with increasing Zr^4+^ content. The reduction in porosity is due to the reduction in crystallite sizes caused by increasing Zr^4+^ content, which results in a denser material structure with fewer voids.

The distances between magnetic ions for the tetrahedral ($L_{A}$) and octahedral ($L_{B}$) sites are determined by calculating the hopping lengths using the following equations [51]:

(7) $$L_{A}=\frac{a\sqrt{3}}{4}\;L_{B}=\frac{a\sqrt{2}}{4}\;$$

Co^2+^ ions predominantly occupy the tetrahedral positions, while Cr^3+^ ions preferentially occupy the octahedral positions. The cation distribution [Co^2+^ ↔ Co^3+^] suggests that the probability of hopping is higher at octahedral sites, as the hopping length $L_{B}$ is shorter than that at the tetrahedral sites, $L_{A}$, as shown in Table 1. The calculated values of $L_{A}$ and $L_{B}$ are consistent with those reported in literature [51]. The increase in both A-site ($L_{A}$) and B-site ($L_{B}$) hopping lengths with increasing Zr^4+^ content is primarily attributed to lattice expansion.

The Scherrer method [52] is widely regarded as the most common technique for estimating crystallite size, utilizing the broadening of the prominent (311) reflection line.

(8) $$D=\frac{k\lambda }{\beta Cos\theta }$$

where k is the shape factor (usually assumed to be 0.9), λ is the wavelength of Cu K_α_ radiation, β is the full width of diffraction reflection at half the maximum intensity, and θ is the diffraction angle.

Another approach is the Williamson–Hall method, which provides more accurate insights into the crystallite size and the contributions of micro-strains observed within the crystal lattice [53].

(9) $$\beta Cos\theta =\frac{k\lambda }{D}+4\varepsilon Sin\theta$$

Here, D and ε correspond to the crystallite size value and microstrain, respectively.

**Figure 3:**
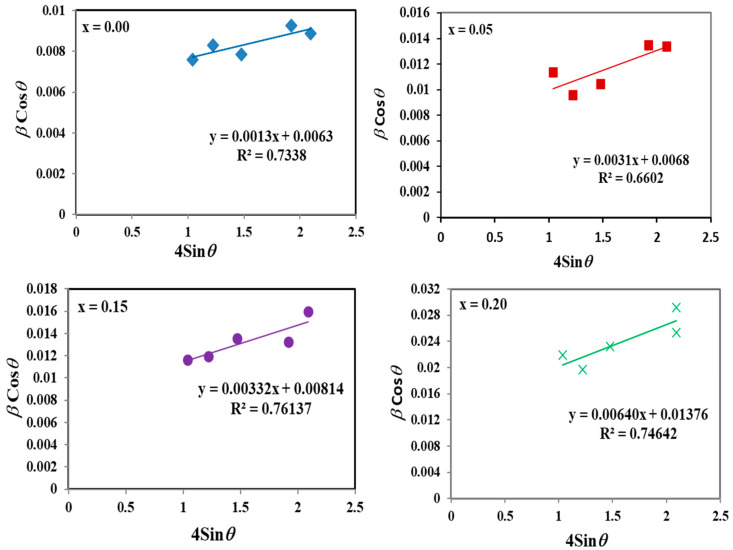
The plots of βCosθ versus 4Sinθ according to the W–H method for CoCr_2−x_Zr_x_O_4_ nanoparticles.

The most intense reflection lines, namely (220), (311), (400), (511) and (400), are selected to create the linear plots. Plotting βCosθ against 4Sinθ allows for estimation of the average crystallite size and strain from the line’s extrapolation and slope, respectively, as shown in Figure 3. Crystallite sizes of CoCr_2−x_Zr_x_O_4_ nanoparticles calculated by the Scherrer equation and the W–H method, along with the strain induced due to crystal imperfection, are listed in Table 1. The crystallite size calculated by both methods shows a consistent tendency, decreasing with increasing Zr^4+^ content, but the obtained values differ. The reduction in crystallite size may be attributed to the substitution of Cr^3+^ ions by Zr^4+^ ions, which introduces lattice distortion due to the differences in ionic radii between Zr^4+^ and Cr^3+^ ions. This distortion can hinder the growth of crystallites, resulting in smaller nanoparticle sizes. The substitution of Zr^4+^ affects the lattice parameters of the CoCr_2_O_4_ structure, impacting both the stability and the growth of the crystallites. An increase in lattice parameters can introduce internal negative stress, further limiting crystallite growth [54]. Strain values increase from 0.0013 to 0.0077 as Zr^4+^ content rises, which correlates with the observed decrease in particle size [55].

**Figure 4:**
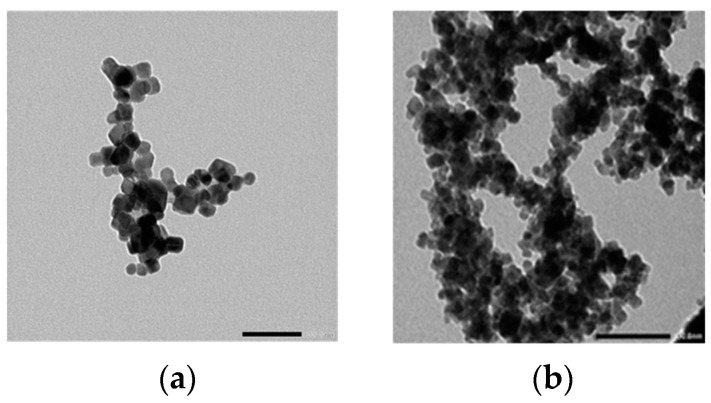

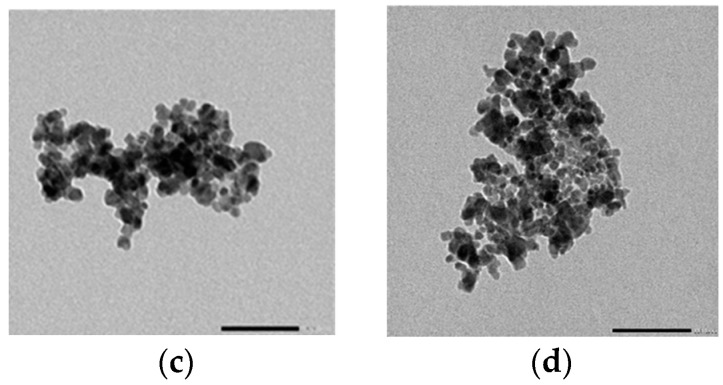
TEM images, with a scale bar of 100 nm, for CoCr_2−x_Zr_x_O_4_ nanoparticles, (**a**) x = 0.00, (**b**) x = 0.05, (**c**) x = 0.10 and (**d**) x = 0.20.

Figure 4a–d show transmission electron micrographs, with a scale bar of 100 nm, for CoCr_2−x_Zr_x_O_4_ at x = 0.0, 0.05, 0.10 and 0.20, respectively. TEM images reveal spherical-like particles, with some agglomeration due to the magnetic nature and interaction between the nanoparticles [55]. Due to the non-uniform particle size, the average size of several particles is calculated. For CoCr_2_O_4_, the mean particle size is approximately 17.0 nm, and it decreases with increasing Zr^4+^ content. The particle size determined by TEM and the crystallite size calculated from the XRD results exhibit a similar trend, both decreasing as the Zr^4+^ content increases, as shown in Table 1. The particle size estimated from TEM images may encompass one or more crystallites, but it is consistently smaller than the crystallite sizes calculated using Scherrer’s equation from XRD spectra. This discrepancy is likely because TEM samples are prepared directly from the reaction solution, while XRD measurements are performed on powdered samples. Aging in the solution influences particle size, making particles more susceptible to aging effects that could increase band gap energy [56]. Thus, the aging process in the reaction solution is likely a key factor contributing to the observed differences between XRD and TEM measurements.

**Figure 5:**
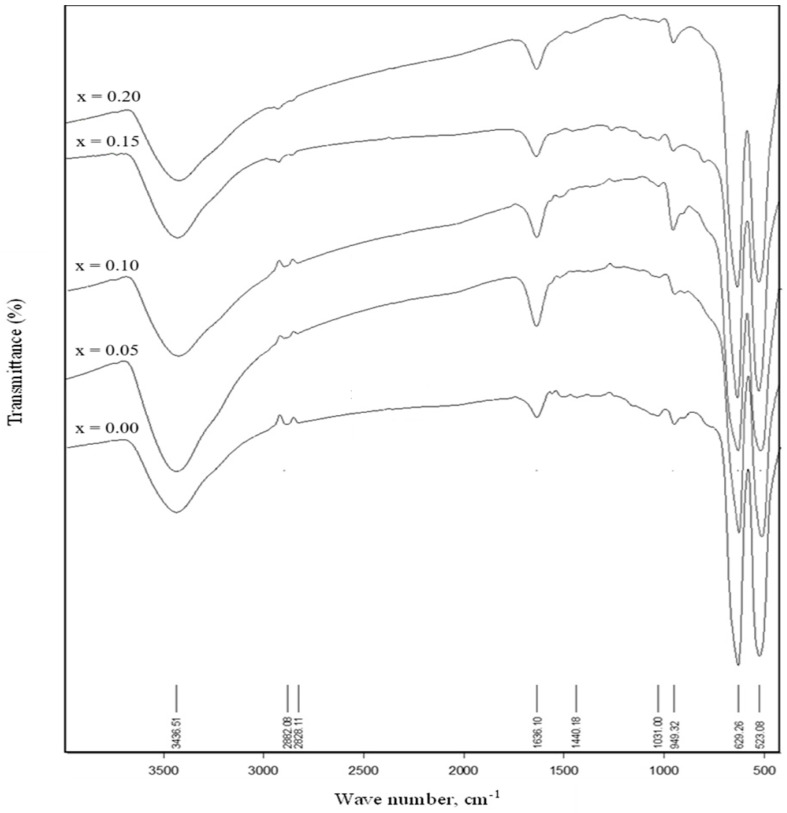
FTIR spectra for CoCr_2−x_Zr_x_O_4_ nanoparticles, 0.00 ≤ x ≤ 0.20.

Spinel structure of multiferroic CoCr_2_O_4_ can be confirmed by infrared spectra (IR), Figure 5 presents the IR spectra of the synthesized nanoparticles within the range of 500–3500 cm⁻^1^. The characteristics of CoCr_2_O_4_ nanoparticles are influenced by the cation distribution at tetrahedral and octahedral sites. Two distinct bands at 629 cm⁻^1^ and 523 cm⁻^1^ confirm the formation of CoCr_2_O_4_ nanoparticles, attributed to the stretching vibrations of Cr^3+^-O^2^⁻ at tetrahedral and octahedral sites, respectively. Additionally, a minor band around 950 cm⁻^1^ corresponds to the Co(II)–O bond, aligning with previous findings [57,58]. A weak absorption band at 1631.58 cm⁻^1^ is due to atmospheric CO_2_, linked to the O–C vibration of the carboxyl group [59]. The final absorption peak observed at 3422 cm⁻^1^ is likely associated with the stretching vibrations of hydroxyl groups (OH) or H–O–H in H_2_O [60]. The values of ν_1_ and ν_2_, summarized in Table 2, show a shift to lower values with increasing Zr^4+^ substitution. The observed reduction in ν_2_ can be explained by the replacement of Cr^3+^ ions with the larger Zr^4+^ ions, which increases the metal-oxygen bond length within the octahedral site, leading to a decrease in the ν_2_ band. It is observed that the ν_1_ values are higher than ν_2_, which can be attributed to the shorter bond lengths in tetrahedral sites, resulting in stronger metal-oxygen bonds and higher vibrational frequencies.

IR data can be used to infer the elastic constants and Debye temperature of the spinel cobalt chromate system.

Force constants of CoCr_2_O_4_ nanoparticles at the tetrahedral ($K_{t}$) and octahedral ($K_{O}$) sites are calculated using the following formula [51]:

(10) $$k=4\pi^{2}c^{2}v^{2}\mu$$

where c is the speed of light (~2.99 × 10 ^10^ cm/s), v is the IR (vibrational) band frequency of the tetrahedral (A) and octahedral (B) sites and μ is the reduced mass of the Cr^3+^ and O^2−^ ions (~2.03 × 10^−23^ g).

Table 2 displays the estimated values of $K_{t}$ and $K_{O}$. It is noted that the force constant at the A-site ($K_{t}$) is greater than that at the B-site ($K_{O}$). The higher force constant at the tetrahedral site (A-site), compared to the octahedral site (B-site) in CoCr_2_O_4_, is due to the shorter bond lengths at the A-site and the stronger metal-oxygen bonds in the tetrahedral coordination (where the cation is surrounded by four oxygen atoms). In contrast, the B-site has an octahedral coordination, with six oxygen atoms surrounding the cation [61].

The lattice constant a and average force constant $K_{av}$ are used to calculate the stiffness constant $C_{11}$ using the following relation [62]:

(11) $$C_{11}=\frac{K_{av}}{a}$$

whereas the stiffness constant $C_{12}$ is given by the following [62]:

(12) $$C_{12}=\frac{C_{11\;}\sigma }{\left(1-\sigma \right)}$$

where σ is Poisson’s ratio and is given by the following [63]:

(13) σ=0.324(1−1.043P)

Table 2 shows that the stiffness constant $C_{11}$ reduces, whereas $C_{12}$ remains nearly constant with increasing Zr^4+^ content in CoCr_2−x_Zr_x_O_4_. The decrease in $C_{11}$ can be attributed to the expansion of the lattice and the increased hopping length, which weaken the interatomic forces. According to Frabtsevich et al. [64], Poisson’s ratio is an indicator of a material’s brittleness or ductility. If Poisson’s ratio is less than 0.26, the material tends to be brittle; if it is greater than 0.26, the material tends to be ductile. It is clear from Table 2 that the ductility of the as-prepared samples increases with increasing Zr^4+^ content. This increase in ductility can be attributed to Zr^4+^ substitution, which influences grain size and the distribution of grain boundaries. A more refined grain structure with well-distributed boundaries enhances ductility, as these boundaries act as barriers to crack propagation.

For isotropic and homogeneous materials like spinel chromates, important elastic constants such as Young’s modulus (E), the bulk modulus (B) and the rigidity modulus (G) are critical in engineering applications.

The values of these moduli for all the samples are calculated by using the following formulas [65,66,67]:

(14) $$Young\;modulus\;\;\;(E)=\frac{\left(C_{11}-C_{12})(C_{11}+{2C}_{12}\right)}{\left(C_{11}+C_{12}\right)}$$

(15) $$Bulk\;modulus\;\left(B\right)\;=\frac{1}{3}\left(C_{11}+{2C}_{12}\right)$$

(16) $$Rigidity\;modulus\;\left(G\right)\;=\frac{E}{2(\sigma +1)}$$

**Figure 6:**
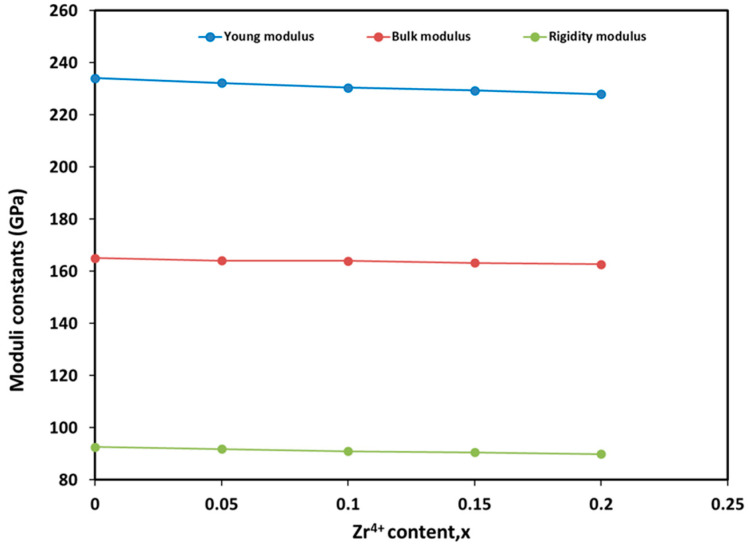
Variation of elastic moduli constants with Zr^4+^ content.

The values of all elastic moduli, *E*, *B* and *G*, are presented in Figure 6. These moduli show decreasing trends as the Zr^4+^ content increases. Comparable results have been reported for Al-substituted nickel ferrites, where the substitution was suggested to enhance interatomic bonding [67]. The decrease in elastic moduli is primarily due to the substitution of Cr^3+^ by Zr^4+^, which causes lattice expansion and weakens atomic bonding, resulting in lower elastic moduli. The increased ductility and decreased rigidity modulus in CoCr_2−x_Zr_x_O_4_ materials make them well-suited for applications in flexible electronics, protective coatings, energy storage components, biomedical implants, and catalytic processes. In energy storage systems, improved ductility allows CoCr_2−x_Zr_x_O_4_ electrodes to better accommodate mechanical stresses during repeated charge-discharge cycles, preventing cracking and increasing the system’s longevity and reliability. Additionally, reduced stiffness aids in distributing mechanical stress evenly, lowering the risk of failure. This also boosts ionic conductivity, leading to faster charge/discharge rates, while facilitating better thermal management by allowing slight deformation under thermal stress, which reduces the risk of thermal cracking in high-power applications.

For coatings, increased ductility enables CoCr_2−x_Zr_x_O_4_ to flex with substrates undergoing mechanical deformation or thermal expansion, maintaining strong adhesion and preventing cracking. This enhanced ductility also improves impact resistance by absorbing and dissipating mechanical forces. The reduced stiffness allows the coating to conform to complex surface geometries, ensuring uniform protection and minimizing residual stresses that could lead to defects or cracks.

In flexible electronics, enhanced ductility allows CoCr_2−x_Zr_x_O_4_ to withstand repeated bending and flexing while maintaining structural integrity and performance, thus improving fatigue resistance. The reduction in stiffness also makes the material easier to bend, which is essential for wearable technology, foldable screens, and other devices that require integration with soft substrates, ultimately enhancing both functionality and user comfort.

**Figure 7:**
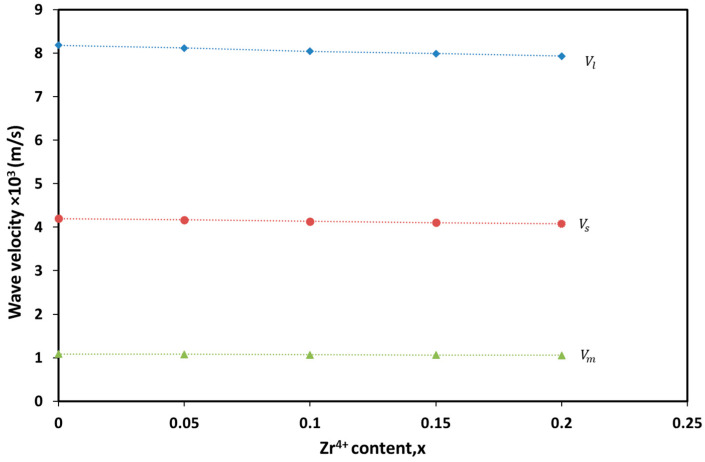
Variation of longitudinal ($V_{l}$), shearing ($V_{s}$) and mean ($V_{m}$) wave velocities with Zr^4+^ content.

The longitudinal elastic wave velocity ($V_{l}$), shear wave velocity ($V_{s}$) and mean wave velocity $V_{m}$ are calculated using the following equations [65]. All velocities show a decreasing trend as the Zr^4+^ content increases, as illustrated in Figure 7.

(17) $$V_{l}={\left(\frac{C_{11}}{\rho_{x}}\right)}^{0.5}$$

(18) $$V_{s}={\left(\frac{G_{0}}{\rho_{x}}\right)}^{0.5}$$

(19) $$\frac{1}{{\;V}_{m}^{3}}=\frac{1}{V_{l}^{3}}+\frac{2}{V_{s}^{3}}$$

where $G_{0}$ is the rigidity modulus with zero pore fraction.

The decrease in shear velocity ($V_{s}$) with increasing Zr^4+^ content is likely due to the larger ionic radius of Zr^4+^ compared to Cr^3+^. This leads to an expansion in the lattice parameters, which in turn reduces the material’s ability to transmit shear waves. Additionally, the substitution of Zr^4+^ (atomic mass ~91.22 u) for Cr^3+^ (atomic mass ~51.996 u) increases the atomic mass, thereby slowing the movement of atoms and reducing the shear velocity.

The Debye temperature is regarded as a fundamental property of solids and can be used to correlate the elastic properties with the thermodynamic properties of solids.

Two methods have been used to determine the Debye temperature: In the first method, the average value of the wavenumbers of bands in the FTIR spectrum is used to calculate the Debye temperature ($\theta_{DIR}$) using Waldron’s formula [68].

(20) $$\theta_{DIR}=\frac{hc\nu_{av}}{k_{B}}$$

where h is Planck’s constant, $k_{B}$ is Boltzmann’s constant, c is the velocity of light and $\nu_{av}$ is the average value of wavenumbers of bands, $\nu_{av}=\left(v_{1}+v_{2}\right)/2$.

The Debye temperature ($\theta_{DA}$) is calculated in the second method using the Anderson Formula [69]:

(21) $$\theta_{DA}=\frac{h}{k_{B}}{\left[\frac{3\rho_{xnN_{A}}}{4\Pi M_{wt}}\right]}^{1/3}V_{m}$$

**Figure 8:**
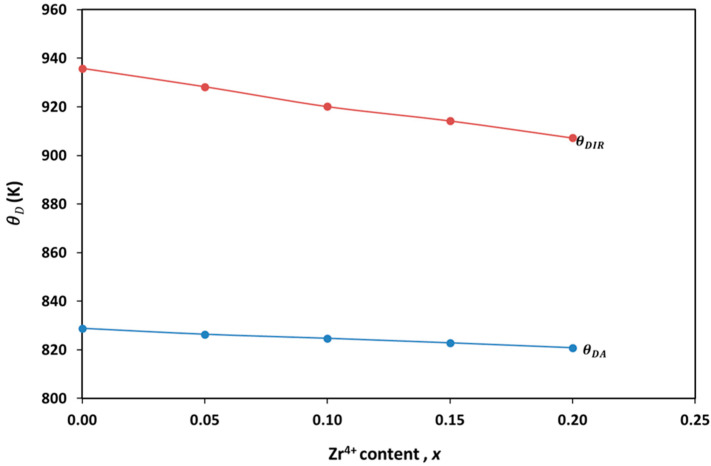
The variation of the Debye temperature $\theta_{DIR}$ and $\theta_{DA}$ with Zr^4+^ content.

Figure 8 demonstrates the good agreement between the Debye temperature computed from the elastic data ($\theta_{DA}$) and infrared data ($\theta_{DIR}$).

As shown in Figure 8, the Debye temperature decreases with increasing Zr^4+^ content. This is due to Zr^4+^’s higher atomic mass compared to Co^2+^ and its tendency to form weaker bonds than Cr^3+^, resulting in reduced lattice stiffness and, consequently, a lower Debye temperature. Similar findings have been reported for Zn-substituted cobalt ferrite [44]. Additionally, the substitution of Cr^3+^ with Zr^4+^ introduces n-type carriers, which may shift the material’s electrical conductivity and create oxygen vacancies to maintain charge neutrality. These additional conduction electrons could interact with phonons, leading to changes in the specific heat and a further reduction in $\theta_{D}$, consistent with specific heat theory [63]. Lower Debye temperatures enhance thermal shock resistance but may also accelerate material degradation due to weaker atomic bonds. As a result, these materials are well-suited for intermediate-temperature applications where both flexibility and moderate thermal management are essential.

**Figure 9:**
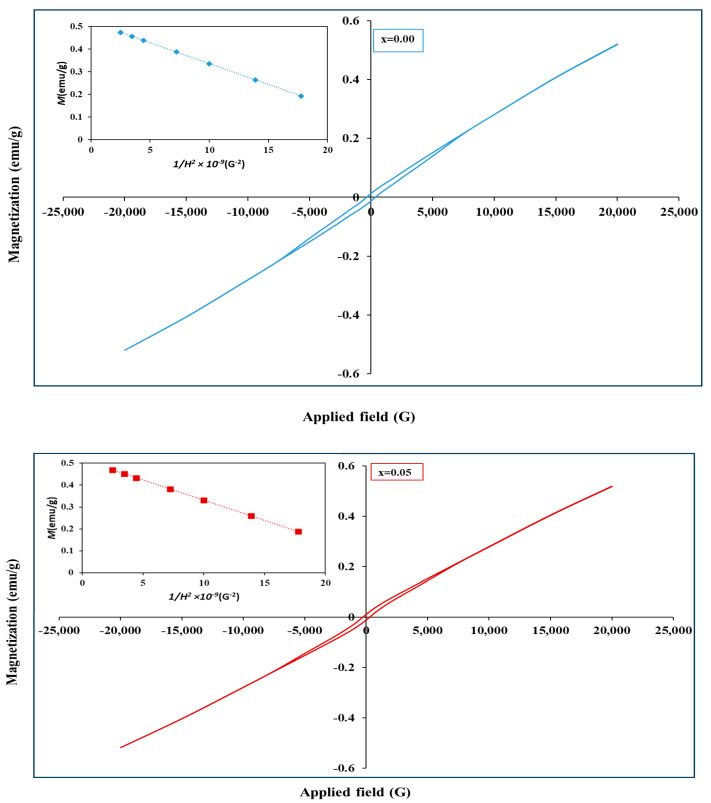

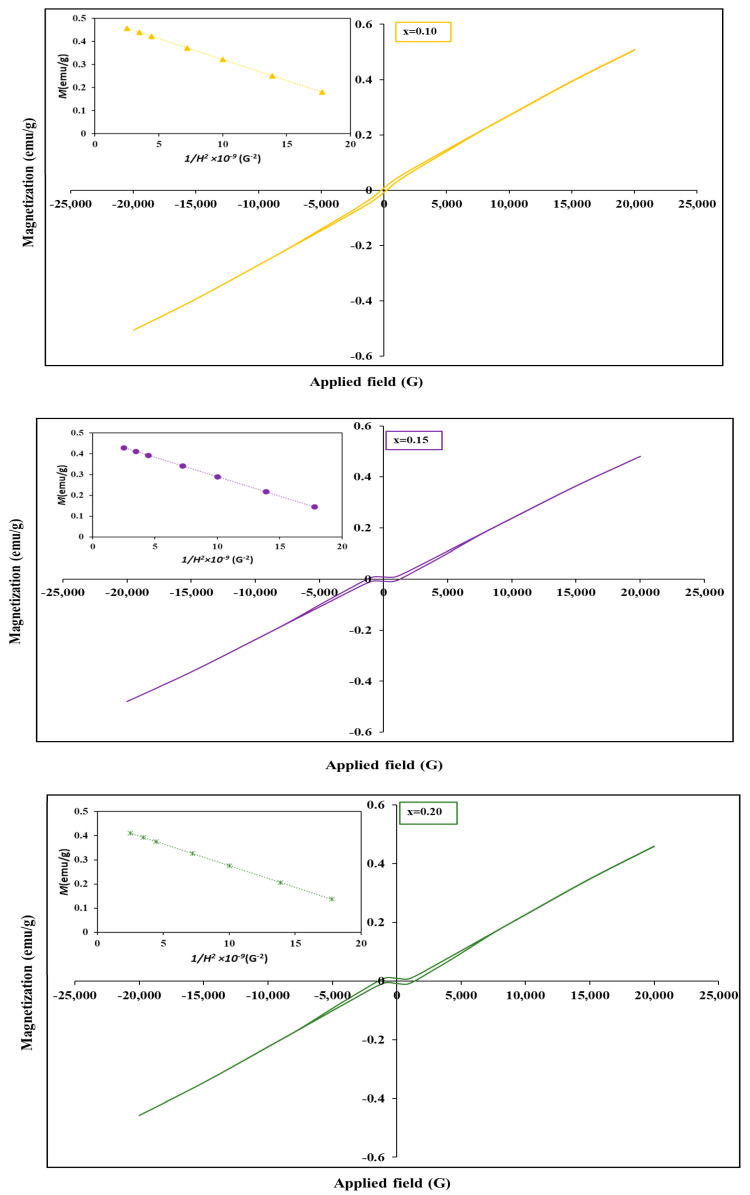
Room temperature magnetic hysteresis loops of BaFe_12−x_Hg_x_O_19_ nanoparticles, x = 0.00, 0.05, 0.10, 0.15 and 0.20. The insets show the plots of M versus 1/$H^{2}$.

Typical plots of the hysteresis loops for various Zr^4+^ doped cobalt chromate nanoparticles samples are shown in Figure 9. CoCr_2_O_4_ exhibits ferrimagnetic behavior at room temperature, as evidenced by its hysteresis loop. The net magnetization arises from the antiparallel alignment of Co^2+^↓ and Cr^3+^↑ ions within the spinel structure. The saturation magnetization ($M_{s}$) of CoCr_2−x_Zr_x_O_4_ is determined by an approximation of Stoner–Wohlfarth (S-W) theory by extrapolating the plot of M versus $1/H^{2}$ to approach zero according to the following equation [70], as displayed in the insets of Figure 9.

(22) $$M=M_{s}\left[1-\frac{\alpha }{H^{2}}\right]$$

The estimated values of $M_{s}$ are displayed in Table 3. It is observed that $M_{s}$ reduces with increasing Zr^4+^ content, whereas the remnant magnetization ($M_{r}$) and coercivity ($H_{c}$) both decrease with increasing x and attain a minimum value at x = 0.10, after which they increase. The reduction in $M_{s}$ is consistent with previous findings for Zr-substituted Sr hexaferrite [71] and BiFeO_3_ [72]. Meanwhile, the increase in coercivity at higher Zr^4+^ content aligns with observations for zirconium-substituted CoFe_2_O_4_ [32]. The decrease in $M_{s}$ is primarily due to the substitution of non-magnetic Zr^4+^ ions for magnetic Cr^3+^ ions, which dilutes the magnetic ion network and weakens the superexchange interactions. Additionally, the larger ionic radius of Zr^4+^ induces lattice distortions, disrupts magnetic interactions and may cause spin canting, further reducing the overall magnetization. The reduction in coercivity with increasing Zr^4+^ concentration is attributed to the decrease in crystallite size. In the single-domain region, coercivity is related to crystallite size by the following equation [73]:

(23) $$H_{c}=g-\frac{C}{D^{2}}$$

where *g* and *C* are constants. However, the increase in coercivity with higher Zr^4+^ content is due to the creation of more pinning sites for magnetic domain walls, caused by increased lattice strain and defects from the substitution of Zr^4+^ ions. This substitution distorts the lattice, enhancing magnetic anisotropy and making domain wall movement more difficult, thus raising coercivity.

The magnetic moment ($\mu_{m}$) is determined from saturation magnetization by utilizing the following formula:

(24) $$\mu_{m}=(M_{wt}\times M_{s})/5585$$

where $M_{wt}$ = molecular weight, and 5585 = magnetic factor.

It is seen from Table 3 that the magnetic moment values gradually decrease from 0.021 to 0.018. The reduction in magnetic moment with increasing Zr^4+^ content is mainly due to the replacement of the magnetic Cr^3+^ ions with non-magnetic Zr^4+^ ions, which lacks unpaired d-electrons and therefore does not contribute to the magnetic moment. Moreover, a high Zr^4+^ concentration can cause disorder, potentially resulting in spin-glass-like behavior that further reduces the observable magnetic moment. CoCr_2−x_Zr_x_O_4_ nanoparticles, with tunable magnetic properties, have a wide range of applications. Their lower coercivity makes them suitable for magnetic data storage, enabling easier writing and reading of data. They can also be used in catalysis for efficient separation processes and to enhance electrochemical performance in batteries and supercapacitors. Additionally, these nanoparticles have potential applications in sensors for detecting magnetic field changes and in composite materials where reduced magnetic strength is beneficial.

## 4. Conclusions

An efficient co-precipitation technique was used to synthesize Zr^4+^-substituted cobalt chromates nanoparticles, CoCr_2−x_Zr_x_O_4_ (0.00 < x ≤ 0.20). X-ray diffraction (XRD) analysis confirmed the formation of a cubic spinel structure in all samples without any secondary phases. The lattice parameter, determined using the Nelson–Riley (N–R) extrapolation method, showed an increasing trend with higher Zr^4+^ content, which can be attributed to the larger ionic radius of Zr^4+^ (0.86 Å) compared to Cr^3+^ (0.755 Å). The broadening of XRD peaks for CoCr_2−x_Zr_x_O_4_ nanoparticles was analyzed using Scherrer’s equation and Williamson–Hall (W–H) analysis, consistent with TEM results. These analyses revealed a gradual decrease in crystallite size as Zr^4+^ content increased. The increase in lattice parameters likely induces internal negative stress, which restricts crystallite growth. Fourier-transform infrared (FTIR) spectra revealed two characteristic absorption bands between 629 cm⁻^1^ and 523 cm⁻^1^, typical of spinel cobalt chromates. Notably, the absorption bands for the Zr^4+^-substituted samples shifted to lower wavenumbers compared to the as-prepared sample. This shift is likely due to the increase in lattice parameter, which leads to a longer metal-oxygen bond in the octahedral site and a consequent reduction in ν_2_. Through infrared spectral analysis, the stiffness constant, elastic moduli, and wave velocity values were determined. A decrease in the stiffness constant and all elastic moduli was observed with the substitution of Cr^3+^ ions by Zr^4+^ ions, attributed to lattice expansion and increased hopping length, both of which weaken interatomic forces. Additionally, the Debye temperature decreased with increasing Zr^4+^ content, linked to the introduction of n-type carriers due to the substitution of Cr^3+^ with Zr^4+^ ions. This process potentially alters electrical conductivity and generates oxygen vacancies to maintain charge neutrality. The magnetic properties of CoCr_2−x_Zr_x_O_4_ (0.00 ≤ x ≤ 0.20) nanoparticles were investigated at room temperature using VSM. Analysis of the M(H) curve revealed that the nanoparticles exhibit nearly ferrimagnetic behavior. Magnetic measurements showed a decrease in saturation magnetization ($M_{s}$) and magnetic moment ($\mu_{m}$) with increasing Zr^4+^ content. This reduction is due to the substitution of non-magnetic Zr^4+^ ions for magnetic Cr^3+^ ions, which dilutes the magnetic ion network and weakens superexchange interactions. Meanwhile, coercivity ($H_{c}$) and remnant magnetization ($M_{r}$) decreased with rising Zr^4+^ content up to x = 0.10 but increased beyond that. This rise in coercivity with higher Zr^4+^ content is attributed to the formation of additional pinning sites for magnetic domain walls, caused by increased lattice strain and defects from Zr^4+^ substitution. This makes domain wall movement more difficult and, as a result, increases coercivity.

## Figures and Tables

**Table 1:** XRD parameters, crystallite size and micro strain of CoCr_2−x_Zr_x_O_4_ nanoparticles, 0.00 ≤ x ≤ 0.20.

Zr^4+^ Content, x	a (Å)	$a_{th}$ (Å)	$\rho_{x}$ (g/cm^3^)	$\rho_{b}$ (g/cm^3^)	*P*%	Hopping Lengths		Scherrer Equation	W-H Method		TEM
						$L_{A}$ (Å)	$L_{B}$ (Å)	*D* (nm)	*D* (nm)	ε × 10^−3^	*L* (nm)
0.00	8.311	8.781	5.251	4.218	19.672	3.598	2.938	18.500	22.01	1.30	16.998
0.05	8.314	8.788	5.291	4.299	18.740	3.600	2.939	14.999	20.42	3.14	11.172
0.10	8.318	8.795	5.328	4.357	18.224	3.602	2.941	14.294	19.53	3.21	9.346
0.15	8.319	8.806	5.372	4.488	16.462	3.602	2.941	11.179	17.03	3.32	7.073
0.20	8.320	8.810	5.415	4.649	14.151	3.603	2.942	9.265	11.91	7.71	6.120

**Table 2:** The absorption bands (ν_1_ and ν_2_), force constant ($K_{t}$ and $K_{O}$) and Poisson’s ratio of CoCr_2−x_Zr_x_O_4_ nanoparticles.

x	ν_1_ (cm^−1^)	ν_2_ (cm^−1^)	$K_{t}\times 10^{5}$ (dyne/cm)	$K_{O}\times 10^{5}$ (dyne/cm)	$C_{11}$ GPa	$C_{12}$ GPa	σ
0.00	629.66	523.08	2.837	1.958	288.538	103.298	0.2636
0.05	626.56	522.79	2.809	1.956	286.627	102.844	0.2641
0.10	625.47	521.57	2.800	1.947	285.3545	103.320	0.2658
0.15	624.81	519.64	2.794	1.932	284.101	102.874	0.2659
0.20	623.74	517.92	2.784	1.919	282.724	102.775	0.2666

**Table 3:** Magnetic parameters of CoCr_2−x_Zr_x_O_4_ nanoparticles.

x	$M_{s}$ (emu/g)	$M_{r}$ × 10^−2^ (emu/g)	$H_{c}$ (G)	$\mu_{m}$
0.00	0.520	1.36	362.07	0.0209
0.05	0.515	1.10	278.92	0.0211
0.10	0.506	0.82	213.25	0.0209
0.15	0.466	0.85	1209.2	0.0194
0.20	0.432	0.93	1357.1	0.0181

## Data Availability

The original contributions presented in the study are included in the article, further inquiries can be directed to the corresponding author.
